# Editorial: Seaweed phylogenetics, evolution, and biogeography

**DOI:** 10.3389/fgene.2024.1429024

**Published:** 2024-06-11

**Authors:** Christophe Vieira, Stefano Draisma

**Affiliations:** ^1^ Research Institute for Basic Sciences, Jeju National University, Jeju City, Republic of Korea; ^2^ Excellence Center for Biodiversity of Peninuslar Thailand, Prince of Songkla University, Hat Yai, Thailand

**Keywords:** phylogeography, macroalgae, seaweed diversification, cryptic diversity, red algae, brown algae

Seaweeds or macroalgae, traditionally perceived as having wide dispersal abilities, have recently undergone molecular scrutiny, revealing nuanced genetic diversity and localized distribution patterns (Díaz-Tapia et al., 2018). In the Research Topic “Seaweed phylogenetics, evolution, and biogeography,” our focus was to delve into the evolutionary and historical processes shaping the diversification and spatial distribution of these marine organisms. Through rigorous genetic analyses and insightful interpretations, the articles in this Research Topic offer significant contributions to our understanding of seaweed evolution and biogeography.

In the first study by Wang et al. (2023), a comprehensive genetic linkage map and quantitative trait loci (QTL) mapping provide insights into the genetic basis of blade morphology in the kelp *Saccharina japonica,* or royal kombu. By pinpointing genomic regions associated with blade traits, the study lays a foundation for marker-assisted selection strategies in seaweed breeding programs, with direct implications for cultivar improvement.

The other studies in this Research Topic describe the phylogeography of red and brown algal species in various regions.

Turning to the northeast Pacific, Gierke et al. (2023) employ a seascape genetics approach to dissect the genetic differentiation of *Nereocystis luetkeana*, or bull kelp. Their study elucidates historical climatic influences and contemporary disturbances, offering valuable insights into the population dynamics of this ecologically significant seaweed species within the Salish Sea region.

Huanel et al. (2024)’s investigation along the Chilean coast unveils the impact of historical vicariance events on the evolutionary history of four species in two genera of the Gigartinales (Rhodophyta). Through sequencing organellar genes, the study reveals strong genetic breaks and sub-structuring patterns, providing valuable insights into the biogeographic dynamics shaping marine ecosystems along the Chilean coastline.

Fontana et al. (2024) delve into the biogeographic intricacies of *Dichotomaria elegans*, a red alga endemic to the northwest Pacific, and its interaction with the Kuroshio Current. Their phylogenetic analyses also reveal cryptic diversity within the circumtropical-warm temperate genus *Dichotomaria*, highlighting the profound influence of ocean currents on genetic divergence and distribution patterns in marine algae.

Lastly, Neiva et al. (2024) shed light on the historical biogeography and genetic cohesion of the wrack *Fucus distichus*, a brown alga that can be found in both the North Pacific and the North Atlantic. Integrating genetic data with ecological niche modeling, the study unveils trans-Arctic dispersal patterns and glacial dynamics, offering critical insights into the evolutionary mechanisms shaping marine biodiversity.

Collectively, these articles represent significant advancements in seaweed science, providing nuanced insights into the evolutionary processes and biogeographic patterns that have shaped the diversity and distribution of marine macroalgae. We extend our appreciation to the authors, reviewers, and editors whose contributions have enriched this Research Topic, fostering future discoveries in this field.
